# Characteristic Factors Affecting Oral Pigmentation in Passive Smoker Children

**DOI:** 10.30476/DENTJODS.2019.81785.0

**Published:** 2020-06

**Authors:** Fahimeh Rashidi Maybodi, Roya Ghafourifard, Mina Mohammad Taheri, Reza Golvardi Yazdi

**Affiliations:** 1 Dept. of Periodontics, Shahid Sadoughi University of Medical Sciences, Yazd, Iran; 2 Dept. of Pediatric, Dental School, Shahid Sadoughi University of Medical Sciences, Yazd, Iran; 3 Dentist, Isfahan, Iran; 4 Student Research Committee, Shahid Sadoughi University of Medical Sciences, Yazd, Iran

**Keywords:** Mouth, Oral health, Pigmentation, Passive smoking, Tobacco, Child

## Abstract

**Statement of the Problem:**

Smoking affects not only smokers themselves, but also the people around them. 700 million children are exposed to second hand tobacco worldwide. One of the adverse effects of being a passive smoker is oral pigmentation.

**Purpose:**

This study was conducted to evaluate the association between smoking of a parent at home and oral pigmentation in children, and the characteristic factors affecting that.

**Materials and Method:**

In this retrospective cohort study, 140 healthy children aged 4 to 10 (mean age= 6.68±1.60), 70 with smoker parent and 70 without smoker parents, were examined for oral pigmentation. Environmental factors were evaluated by asking the parents to fill a questionnaire. Data were analyzed using Chi-square test, Fisher's exact test, Logistic regression, and Spearman scale.

**Results:**

There was a meaningful relationship between having a smoker parent and oral pigmentation (*p*= 0.0001). Spearman’s correlation showed parents' duration of cigarette smoking and the number of cigarettes per day could meaningfully affect the severity of oral pigmentation (R=0.329). The study did not find a statistical relationship between oral pigmentation in passive smoking and gender or house area.

**Conclusion:**

Children exposed to secondhand tobacco are at more risk for oral pigmentation. Its severity depends on duration of cigarette smoking and the number of cigarettes per day.

## Introduction

Smoking affects not only the smokers themselves but also the people around them. According to world health organization (WHO), 700 million children (40% of children) are exposed to second hand tobacco worldwide [ 1 ]. Studies have shown various adversities for being a passive smoker in children such as respiratory problems, low birth weight, developmental disabilities, increased risk for chronic obstructive pulmonary disease (COPD), and cancers such as lung cancer [ 1 - 3 ]. Smokers develop areas of darkened mucosa in their oral cavity called hyperpigmentation, which are the excessive sedimentations of melanin [ 4 - 5 ]. Although most studies have shown a relationship between oral pigmentation and being a passive smoker, there is controversy on the effect of gender, duration of exposure, and house area [ 4 , 6 - 11 ].

The purpose of this study was to survey the association between smoking of parents at home and oral pigmentation in children, concerning the affecting characteristic factors.

## Materials and Method

### Design and sampling

The present study is a retrospective (historical) cohort study. Considering oral pigmentation prevalence in passive smoker children (62%) and non-passive smoker children (38%) in Yasear *et al*. study [ 12 ], the sample size for each group was calculated to be *n*=70 using the following equation (significance level= 0.05, power test = 0.80): 

$n=\frac{{\left(Z_{\mathrm{\alpha /2}}+Z_{\beta }\right)}^{2}[P_{1}(1-P_{1})+P_{2}(1-P_{2})]}{{\left(P_{1}-P_{2}\right)}^{2}}$

A total of 70 children (4.5 to 10 years old) referred to the Pediatric Department of Yazd Dental School (from October to December 2017) whom at least one of their parents or caregivers were smoker at home were selected as the case group. In our study, a person who had consumed at least ten cigarettes during the last month in the presence of their child was considered as smoker. This data was acquired by asking the parents. Seventy children attending pediatric department of Yazd Dental School were selected as the control group from those with non-smoker parents. The control group had the same age range as the case group. The inclusion and exclusion criteria were applied to both groups as follows. All children were psychologically and physically healthy (based on questionnaires) and did not take any medications, such as Bismuth and so on, which would have effects on the pigmentation of periodontium.

In this study, the inner side of arm was chosen to determine the skin color [ 7 ]. MY cream (MY, Iran) was used as an indicator to classify the skin color. Five tinted cream numbers were used from one to five. Numbers 1 to 3 were considered as light skin complexions, and numbers 4 and 5 as dark skin complexions. There is a higher possibility of occurrence of physiological pigmentation in people with darker skin color, therefore, dark-colored skin subjects were excluded and replaced [ 3 ].

### Evaluating environmental factors 

A form was designed containing information on the study and asking about the parents' consent on participating. After the consent was given, a questionnaire including questions on demographic information (child’s age and gender), parents’ education and child’s medical history (presence or history of hyperthyroidism, hypothyroidism, adrenal hyperthyroidism, adrenal insufficiency, allergies, abnormal hemorrhages, vitamin B12 deficiency, Peutz-Jeghers syndrome) was given to parents or caregivers. The surface area of their house was asked with the two options, below and over 100 square meters. This variable was analyzed only in the case group to see how the house area affects the children who have a smoker parent. During the sampling process, the control group was selected to be similar to cases on the distribution of age, gender, and skin color.

### Examination 

The size of pigmentations was measured by a graded periodontal probe and was documented along with their locations in oral cavity (mucosa, lips, tongue, and gums). A classification was used to determine severity of the lesions defined as (1) mild (0.5-1cm), (2) moderate (1-2cm), and (3) severe (more than 2cm or presence of multiple sites of pigmentation).

### Statistical analysis

Data were collected, coded, and entered to the computer. They were analyzed using SPSS23 software. Qualitative variables were analyzed via Chi-square test. Pigmentation severity was considered an ordinal variable; therefore, Spearman correlation was used for its analysis. 

## Results

A sample of 140 children with an average age of 6.68± 1.60 years was randomly selected. Out of the 140 samples examined, only two had mild allergies and did not use any medications. These two samples were excluded and replaced so that the effect of systemic diseases variable was excluded from the study. After reviewing the data, the following results were obtained:

Oral mucosal pigmentation, regardless of the effect of passive smoking on children, was observed in 50.7% of the subjects. 20.7% of them had mild pigmentation, 20.7% had moderate pigmentation and 3.9% had severe pigmentation. Therefore, the least frequent type of pigmentation was severe (Table 1).

### Gender

The sample was consisted of 69 girls and 71 boys. There was no significant relationship between gender and the presence of pigmentation (Table 1; *p*= 0.280).

**Table 1:** Relationship between gender and the presence of pigmentation

	Gender		Total n(%)
	Male	Female	
None n(%)	33 (46.5)	36 (52.2)	69 (49.3)
Mild n(%)	19 (26.8)	10 (14.5)	29 (20.7)
Moderate n(%)	12 (16.9)	17 (24.6)	29 (20.7)
Sever n(%)	7 (9.8)	6 (8.7)	13 (9.3)
Total n	71	69	140

*p* Value=0.280

### Smoker parent 

The case group consisted of 70 children with a smoker parent, all of whom their fathers were the smoker. Of these 70 patients, 31.4% had no pigmentation, 34.3% had mild pigmentation, 20% had moderate pigmentation, and 14.3% had severe pigmentation. In total, 48 had oral pigmentation. The control group consisted of 70 children without a smoker parent or caregiver. A total of 67.1% had no pigmentation, 7.2% had mild pigmentation, 21.4% had moderate pigmentation, and 4.3% had severe pigmentation. In total, 23 children with oral pigmentation were detected in the control group. There was a significant statistical difference between the presence of oral pigmentation and having a smoker father (Table 2; *p*= 0.0001). Relative risk of passive smo--king for the occurrence of oral pigmentation in passive smoker children was 2.10 (C.I. = 95%: 1.45 – 3.05).

**Table 2:** Relationship between the presence of oral pigmentation and having a smoker father

	Smoker Parent		Total n (%)
	+	-	
None n (%)	22 (31.4)	47 (67.1)	69 (49.3)
Mild n (%)	24 (31.4)	5 (7.2)	29 (20.7)
Moderate n (%)	14 (20)	15 (21.4)	29 (20.7)
Severe n (%)	10 (20)	3 (4.3)	13 (9.3)
Total n	70	70	140

*p* Value= 0.0001

### Oral site

In all cases, the ​​involvement area was observed in the anterior region of the mouth and the labial gingiva.

### House area

There was no statistically significant difference, and no meaningful relationship between house size and the occurrence of children oral pigmentation (Table 3; *p*= 0.2). Data are presented in Table 3.

**Table 3:** Relationship between house size and the occurrence of children oral pigmentation

	House Area		Total n (%)
	Over 100	Under 100	
None n (%)	13 (38.24)	9 (25)	22 (31.43)
Mild n (%)	12 (35.29)	12 (33.33)	24 (34.29)
Moderate n (%)	6 (17.65)	8 (22.23)	14 (20)
Severe n (%)	3 (8.82)	7 (19.44)	10 (14.28)
Total n	34	36	70

*p* Value= 0.2

### Age

The relationship between age and oral pigmentation was statistically significant (Table 4; *p*= 0.001). Spearman's correlation coefficient for the age variable was 0.282. 

**Table 4:** Spearman correlation for age, duration of smoking and number of cigarettes per day

	Spearman Correlation	*p* Value
Age	0.282	0.001
Duration of smoking	0.371	0.0001
Number of cigarettes per day	0.358	0.0001

Given the positive number obtained, the age was directly related to the pigmentation, meaning that older children presented more oral pigmentation.

### Duration of smoking

This variable had a significant relationship with children’s oral pigmentation (Table 4; *p*= 0.0001). Spearman correlation coefficient of duration of smoking (the number of years the parent has been smoking) was 0.371. Therefore, duration of consumption was directly related with the pigmentation. 

### Number of cigarettes per day

There was a statistically significant difference and this variable was related to pigmentation (Table 4; *p*= 0.0001). Spearman's correlation coefficient in relation to this variable was 0.358. Therefore, it also had a direct relationship with the two other variables. 

## Discussion

This study aimed to examine the relationship between smoking of a parent at home and oral pigmentation in children and the characteristics affecting that. Studies on effects of passive smoking on oral pigmentation have been conducted on different target groups. Moravej-Salehi *et al*. [ 11 ] have investigated its effects on non-smoking women. Hajifattahi *et al*. [ 6 ], Hanioka *et al*. [ 4 ], Yasear *et al*. [ 12 ], Sridharan *et al*. [ 10 ], similar to current study, investigated its effects on children with a smoker parent. All these studies have confirmed the positive relationship between passive smoking and oral pigmentation.

Hanioka *et al*. [ 4 ] and Yasear *et al*. [ 12 ] also matched age, and gender in their case and control groups and have demonstrated the positive relation between severity of the lesions and age. Other studies have not considered this variable. 

As mentioned before, by using Spearman’s correlation analysis, a positive relation between oral pigmentation and the children’s exposure to second hand tobacco was found. Some studies on effects of active smoking have shown the same results [ 8 , 13 ] but unfortunately, studies on effects of passive smoking have not considered these variables [ 6 , 11 ]. In this study, the subjects were in complete physical and psychological health, not using any medications that induce pigmentation. Madani *et al*. [ 7 ] and Hanioka *et al*. [ 4 ] have not excluded these intervening variables.

Using MY cream, skin complexions were matched and subjects with darker skins were not included in the study. Haji-Fattahi *et al*. [ 6 ], Moravej-Salehi *et al*. [ 11 ] and Sridharan *et al*. [ 10 ] employed similar methods to eliminate the possibilities of physiological pigmentation. However, Madani *et al*. [ 7 ] and Yasear *et al*. [ 12 ] have not examined skin color in their studies.

In this study, lesions were categorized by size. Periodontal probe was used to measure the size of the lesions. The examinations were conducted by only one clinician to ensure the objectivity of study. Madani *et al*. [ 7 ] classified lesions using Dummett oral pigmentation index (DOPI). Hanioka *et al*. [ 4 ] also measured the size; however, they used photos of the lesions, not direct examination. Other previous studies, only reported the presence of lesions with no attempt to measure the size of the lesions [ 11 - 12 ]. Nevertheless, in this study, we classified lesions based on size. Concerning involved oral sites, most lesions were found in the anterior areas of maxilla and mandible. Other studies that investigated site of the lesions have had the same findings [ 6 , 11 ].

Despite this study, Moravej-Salehi *et al*. [ 11 ] were able to find a meaningful relationship between oral pigmentation in passive smokers and surface area of their houses. This can be due to the age difference of the subjects. Children are more dependent to their parents and keep least distance from them. Their subjects included adult women who had smoker husbands whom may keep away from their husbands while they are smoking [ 11 ]. 

It must be mentioned that smoking has different adverse effects on the consumer’s and their families' health. This study has investigated just one of the many harmful effects of children's passive smoking.

## Conclusion

Children exposed to secondhand-tobacco regardless of their gender are at more risk to develop oral pigmentation. Investigating factors affecting the severity of pigmentations, this study found that parents' duration of cigarette smoking and the number of cigarettes per day have a meaningful relationship with their child's oral pigmentation. As for the extent of house area, this study could not find a relationship. In conclusion, prohibition of smoking in the presence of children might prevent oral pigmentation as well as other adverse effects of being a passive smoker.
